# Draft genome sequence of a clinical *Staphylococcus epidermidis* strain, NGCE-2436, from Bangladesh

**DOI:** 10.1128/mra.00189-26

**Published:** 2026-07-10

**Authors:** Md. Mashiur Rahaman, Fahad Khan, Sumia Jahan Shatu, Abdus Sadique, Jahidul Alam, Farhana Shahid Labanya, Kamrul Islam, Fariza Shams, Anica Tasnim Protity, Ayesha Sharmin, Md. Mahbubur Rahman, Md. Mainul Hossain, Maqsud Hossain

**Affiliations:** 1 NSU Genome Research Institute (NGRI), North South University 54495, Dhaka, Bangladesh; 2 Department of Biochemistry and Microbiology, School of Health and Life Sciences, North South University 54495, Dhaka, Bangladesh; 3 Department of Chemistry, Bangladesh University of Engineering and Technology 61750, Dhaka, Bangladesh; 4 Department of Pharmaceutical Science, School of Health and Life Sciences, North South University, Dhaka, Bangladesh; Fluxus Inc., Sunnyvale, California, USA

**Keywords:** *Staphylococcus epidermidis*, whole-genome sequencing, antimicrobial resistance

## Abstract

We report the draft genome sequence of *Staphylococcus epidermidis* NGCE-2436 from a nasal swab of a flu-infected patient in Dhaka, Bangladesh. Illumina MiSeq sequencing produced a 2.47 Mb assembly (81 contigs; 31.87% GC) with 2,374 predicted coding sequences, supporting resistome and pathogenicity assessment.

## ANNOUNCEMENT

*Staphylococcus epidermidis* is a common skin commensal and one of the most frequently isolated coagulase-negative staphylococci (CoNS) in clinical infections, particularly associated with indwelling devices and immunocompromised patients (1, 2). Its role in polymicrobial respiratory infections and its increasing antibiotic resistance profile underscore the need for genomic surveillance (3).

The strain NGCE-2436 was isolated from the nasal swab of a flu-infected patient at the Dhaka Central International Medical College and Hospital (DCIMCH), Dhaka, Bangladesh, in December 2024. The swab sample was enriched overnight in Luria-Bertani (LB) broth at 37°C, and then streaked on Mannitol Salt Agar (MSA) and incubated overnight at 37°C. A presumptive single pink colony was picked and inoculated in LB broth media and incubated overnight at 37°C for genomic DNA extraction using the QIAamp DNA Mini Kit (Qiagen, Germany) following manufacturer’s protocol with prior lysozyme treatment. Species identity was confirmed via PCR using the gseA primers (gseA-F: 5′-GGCAAATTTGTGGGTCAAG-3′ and gseA-R: 5′-TGGCTAATGGTTTGTCACCA-3′). PCR was performed with an initial denaturation at 95°C (5 min), followed by 30 cycles of 95°C (30 s), 53°C (30 s), and 72°C (50 s), and a final extension at 72°C (10 min) (4); PCR product was then visualized on a 1.5% agarose gel (amplicon size 193 bp).

For whole-genome sequencing, library preparation was done using the Nextera XT DNA Library Preparation Kit (Illumina, USA), and sequencing was performed using the Illumina MiSeq platform using the MiSeq Reagent Kit v3 with 2 × 150 bp paired-end approach following manufacturer’s instruction. FastQC v0.12.0 (5) analysis revealed a total of 979,625 paired-end reads were generated (~72.8 Mb per file). Adapter trimming and quality filtering (AVGQUAL ≥30) with Trimmomatic v0.39 (6) produced ~708,886 high-quality reads per pair.

*De novo* genome assembly with SPAdes v3.15.4 (7) (coverage cutoff ≥30×; contig length ≥200), produced a genome with 81 contigs and a total length of 2,471,213 bp, final genomic coverage of 58.29× (N50 67,267 bp, GC content 31.87%). Assembly quality was assessed using QUAST v5.0.2 (8). Genome completeness assessment using BUSCO v5.8.0 (9) showed 99.3% completeness. The average nucleotide identity (ANI) (10) with *S. epidermidis* reference genome GCF_000011925.1 was 99.29%.

Genome annotation with NCBI Prokaryotic Genome Annotation Pipeline (PGAP) v6.10 (11) predicted a total of 2,374 genes, of which 2,261 are protein-coding genes, 3 rRNAs, and 25 tRNAs. MLST-2.0 analysis (12) assigned the strain to sequence type ST16.

ResFinder v4.7.2 (13) (Resfinder database v2.5.1) detected genes conferring resistance to aminoglycosides (aph(3′)-III, ant(6)-Ia), beta-lactams (mecA, blaZ), macrolides (msr(A), mph(C)), streptogramins (msr(A)), and fusidic acid (fusB). PathogenFinder (14) predicted human pathogenic potential with a mean score of 0.68 across neural networks. All tools were used at their default parameters, except where otherwise mentioned.

Strain NGCE-2436, isolated from a polymicrobial sample of a flu patient, provides key genomic insights into secondary bacterial dynamics during viral infection. These data expand the genomic knowledge of clinical *S. epidermidis* strains circulating in South Asia and highlight the importance of continuous monitoring of resistance determinants in commensal pathogens.

## Data Availability

The whole-genome sequence of *S. epidermidis* strain NGCE-2436 has been deposited in GenBank (JBQWSR000000000). Raw reads are available in the NCBI SRA (SRR36783064) under BioProject PRJNA1320989 and BioSample SAMN51192840.
